# Rationale for a New Low-Dose Triple Single Pill Combination for the Treatment of Hypertension

**DOI:** 10.5334/gh.1283

**Published:** 2024-02-14

**Authors:** Anthony Rodgers, Abdul Salam, William Cushman, Asita de Silva, Gian Luca Di Tanna, Sonali R. Gnanenthiran, Diederick Grobbee, Krzysztof Narkiewicz, Dike Ojji, Suzanne Oparil, Neil Poulter, Markus P. Schlaich, Aletta E. Schutte, Wilko Spiering, Bryan Williams, Jackson T. Wright, Paul Whelton

**Affiliations:** 1 The George Institute for Global Health, University of New South Wales, Australia; 2 The George Institute for Global Health, University of New South Wales, India; 3 Prasanna School of Public Health, Manipal Academy of Higher Education, India; 4 University of Tennessee Health Science Center, USA; 5 University of Kelaniya, Sri Lanka; 6 University of Applied Sciences and Arts of Southern Switzerland, Switzerland; 7 University Medical Center Utrecht, Utrecht University, The Netherlands; 8 Medical University of Gdańsk, Poland; 9 University of Abuja, Nigeria; 10 University of Alabama at Birmingham, USA; 11 Imperial College, London, UK; 12 The University of Western Australia, Australia; 13 University College London, UK; 14 University Hospitals Cleveland Medical Center, Case Western Reserve University, USA; 15 Department of Epidemiology, Tulane University School of Public Health and Tropical Medicine, New Orleans, USA

**Keywords:** hypertension, polypill, blood pressure, single pill combination, fixed dose

## Abstract

Two recent large trials showed the potential of single pill combinations (SPCs) with ≥3 low-dose components among people with hypertension who were untreated or receiving monotherapy. In both trials, these ‘hypertension polypills’ were superior to usual care, achieving >80% BP control without increasing withdrawal due to side effects. However, there are no such products available for prescribers. To address this unmet need, George Medicines developed GMRx2 with telmisartan/amlodipine/indapamide in three strengths (mg): 10/1.25/0.625, 20/2.5/1.25; 40/5/2.5. Two pivotal trials are ongoing to support FDA submission for the treatment of hypertension, including initial treatment. These assess efficacy and safety of GMRx2 compared to: placebo, and each of the three possible dual combinations. Regulatory submissions are planned for 2024, with the aim of providing access to GMRx2 in developed and developing regions. Wider implementation of GMRx2-based treatment strategies will be guided by further research to inform access and appropriate scale up.

## Burden of Hypertension and Current Treatment Gaps

High blood pressure (BP) is a leading cause of the global disease burden and less than 1 in 4 treated individuals achieve recommended BP goals [1,2,3,4]. Globally, most treated patients only receive monotherapy, despite broad consensus that the large majority of patients require multiple antihypertensive drugs [3,4,5,6]. In the United States of America (USA), about 40% of treated patients receive monotherapy, and this has not changed substantially in recent decades [7]. Given the recommendations in recent guidelines for lower BP targets for many patients, the need for effective, tolerable and affordable therapy is even more imperative for the initial treatment of hypertension.

## Potential Role of Single Pill Combinations, Including Low-Dose Options

Recent hypertension guidelines, including the 2018 European Society of Cardiology/European Society of Hypertension (ESC/ESH) Guideline, [4] the 2017 American College of Cardiology/American Heart Association (ACC/AHA) Guideline, [3] the 2020 International Society of Hypertension Guideline [8] and the 2021 WHO Hypertension Guideline [9] now suggest combination blood pressure (BP)-lowering of two drugs as initial treatment for many or most patients. These recommendations were based on evidence that dual combinations compared to monotherapy achieve better BP control, improve adherence and reduce therapeutic inertia, without important increases in adverse effects [10,11,12,13]. In recent years there has been interest in low-dose combination therapy with three or more drugs, with evidence indicating that low-dose treatment of multiple drug classes may achieve greater BP control without increasing adverse effects:

- Much of the BP-lowering efficacy of any drug in monotherapy is achieved at low doses and efficacy dose-response curves are typically shallow above one quarter of the standard dose [14,15].
- At low doses there are few or no adverse effects, but for many classes adverse effects rise steeply and steadily as the dose increases [14,15].
- The incidence of idiosyncratic reactions (such as anaphylaxis) to BP-lowering drugs is so low that the risks for a patient simultaneously taking three drugs is acceptably low [15].
- There is additivity of effects across drug classes that target different pathophysiological pathways [10,14,15].
- There is clear evidence of greater reduction in cardiovascular events with greater BP reduction [16,17].

**Figure 1:**
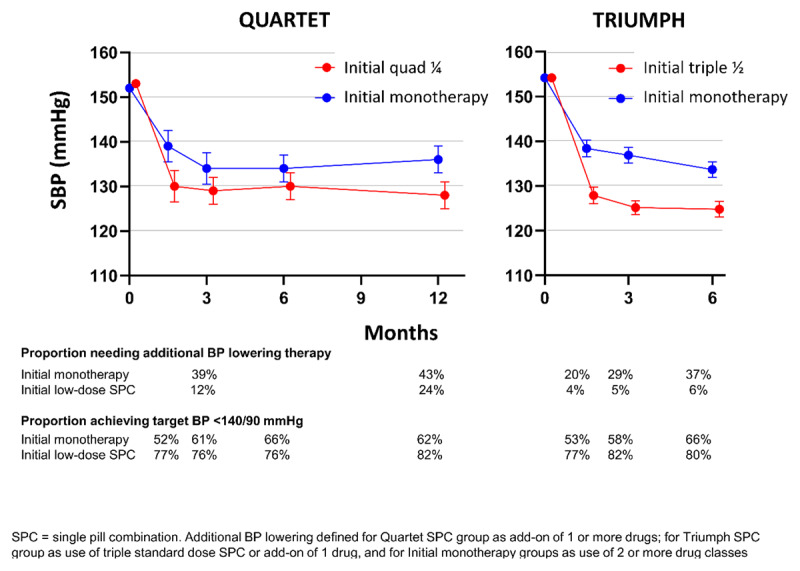
Summary of the QUARTET [22] and TRIUMPH [20] trials of low-dose combination vs usual care for initial/early treatment of hypertension.

Large and tolerable reductions in BP have been demonstrated in four short-term (4–12 weeks) trials of triple or quadruple low-dose SPC therapy (Table 1) [18,19,20,21]. These trials provided the rationale for the triple pill vs usual care management for patients with mild-to-moderate hypertension (TRIUMPH) [20] and the quadruple ultralow dose treatment for hypertension (QUARTET) [22] trials among patients with hypertension, who were either untreated or managed with monotherapy. TRIUMPH was a six-month open label randomized controlled trial (RCT) conducted in Sri Lanka that compared initial or early low-dose triple single pill combination (SPC) (telmisartan 20 mg, amlodipine 2.5 mg, chlorthalidone 12.5 mg) with usual care in adults with uncontrolled hypertension [20]. The QUARTET was a 12-week double blinded RCT in 591 Australian adults with uncontrolled hypertension that compared treatment starting with an ultra-low-dose combination (containing irbesartan 37.5 mg, amlodipine 1.25 mg, indapamide 0.625 mg, and bisoprolol 2.5 mg) to guideline-recommended treatment starting with standard dose monotherapy (irbesartan 150 mg). As shown in Figure 1, both trials showed large improvement in BP control achieved quickly and sustained over 6–12 months without usual care group ‘catch-up’ despite considerably higher rates of add-on BP treatment in the usual care group. Furthermore, the usual care groups had superior levels of care, with lower treatment inertia rates and higher BP control rates than are generally seen in normal clinical practice.

**Table 1:** Short-term RCTs of triple or quadruple low-dose combination therapy.


TRIAL	COMPARISON	BP REDUCTION	TOLERABILITY

Mahmud et al 2007 [18]	4 × ¼ dose (amlodipine 1.25 mg, atenolol 12.5 mg, bendroflumethiazide 0.625 mg, captopril 25 mg, n = 22) vs each at standard dose (n = 86)	13/8 mmHg (p < 0.001)	No SAEs or treatment withdrawals

Wald et al 2012 [19]	3 × ½ dose (amlodipine 2.5 mg, losartan 25 mg, hydrochlorothiazide 12.5 mg) vs. placebo (n = 86 crossover)	18/10 mmHg (P < 0.001)	No SAEs or treatment withdrawals

Chow et al 2017 [21]	4 × ¼ dose (irbesartan 37.5 mg, amlodipine 1.25 mg, HCTZ 6.25 mg and atenolol 12.5 mg) vs. placebo (n = 21 crossover)	22/13 mmHg p < 0.001	No SAEs or treatment withdrawals

Hong et al 2020 [54]	amlodipine, losartan and chlorthalidone at 3 × ½ dose, 3 × ⅓ dose and 3 × ¼ dose (n = 107) vs. placebo (n = 36) for 8 weeks	17/9, 20/10 and 14/8 mmHg, respectively (all p < 0.01)	No SAEs, 1 treatment-related withdrawal


## Rationale for a New Triple Single Pill Combination

Currently available triple SPCs are only indicated for substitution among patients already taking all the three-component drugs, or as an add-on/switch therapy among patients not adequately controlled on two of the component drugs [23,24,25]. None of the available triple SPCs are available in low-dose strengths, nor indicated for initial treatment. Furthermore, there are no existing SPC products on the market with low-doses of an angiotensin receptor blocker (ARB), calcium channel blocker (CCB) and a thiazide-like diuretic. To address this unmet need, George Medicines (GM) developed a new SPC containing telmisartan, amlodipine and indapamide, named GMRx2, in three strengths (Table 2).

**Table 2:** GMRx2 strengths.


GMRx2 DOSE VERSION	STANDARD DOSES OF COMPONENT DRUGS	TELMISARTAN/AMLODIPINE/INDAPAMIDE DOSES (MG)

1	¼	10/1.25/0.625

2	½	20/2.5/1.25

3	1	40/5/2.5


The rationale for the choice of the individual drug components of GMRx2 is based on several considerations [26]. Hypertension guidelines recommend use of drugs from the four following antihypertensive drug classes: ACEI, ARB, CCB, or thiazide/like diuretics for first-line therapy of adults with hypertension. ARBs are better tolerated than ACEIs and are less likely to cause angioedema among black patients [27], with direct randomized comparisons suggesting comparable efficacy in reducing CV events [28,29,30]. Telmisartan has the longest half-life (~24 hours) of all ARBs [31]. Among CCBs, amlodipine has been used most commonly in CV outcome trials, has little potential for drug interactions and has a long half-life (30–50 hours) [17,32]. Low-doses of the thiazide-like indapamide provide good BP reduction with low side effect profiles [15,33,34,35,36], has a half-life of 16 to 18 hours [37], and is widely available internationally.

The systolic BP (SBP)-lowering efficacy of GMRx2 strengths 1, 2 and 3 (Table 2) from a baseline SBP of 150 mmHg are expected to be approximately 13 mmHg, 18 mmHg and 25 mmHg, respectively [38]. This represents a much greater and clinically important BP reduction in comparison to standard-dose monotherapy, which reduces SBP compared with placebo by only 8–9 mmHg on average, with each doubling of dose conferring only a 1–2 mmHg incremental SBP reduction [10,15]. Dual combinations typically show only a 1–2 mmHg difference between neighboring drug doses and an SBP reduction of around 20 mmHg at maximal dose [10,15,38]. The rationale for developing GMRx2 is, therefore, in line with USA Food & Drug Administration (FDA) observations:

‘Over the last decade, the Agency has actively discouraged antihypertensive monotherapy and combination doses with effects that were very close together, considering them a nuisance to physicians seeking to get patients to goal [39]’

## Summary of the GMRx2 Trial Program

The main design features of the two phase III trials for FDA approval of GMRx2 are outlined in Table 3, with further details reported on clinicaltrials.gov [40,41]. Both trials are expected to be completed in 2023. The overall clinical program is directed by an independent steering committee of international experts (Table 4) and an independent data and safety monitoring board (Table 5).

**Table 3:** Summary of characteristics of the phase III efficacy and safety trials of GRMx2.


TRIAL	DESIGN	PARTICIPANT KEY ELIGIBILITY FOR RANDOMIZATION	KEY EXCLUSION CRITERIA	RANDOMIZED ALLOCATION	INTERVENTION, COMPARATOR	DURATION OF RANDOMIZED TREATMENT AND FOLLOW UP	PRIMARY OUTCOME	PRIMARY SAFETY OUTCOME	SAMPLE SIZE, POWER

GMRx2 ACT	Randomized, double-blind, active-controlled, parallel-group, international multi-center	Adults on ≤3 BP-lowering drugs, adhering to and tolerating GMRx2 dose 2 run-in and post run-in home SBP 110-154 mmHg	Receiving 4 or more BP-lowering drugs. Contraindication to the individual components of the GMRx2	2:1:1:1 ratio to GMRx2 or one of the three comparators of dual combinations	Intervention: GMRx2 dose 2 Comparators: telmisartan 20 + amlodipine 2.5, Or telmisartan 20 + indapamide 1.25, Or amlodipine 2.5 + indapamide 1.25 for 6 weeks, forced uptitration to double dose for 6 weeks	12 weeks + 4 weeks safety follow up	Difference in change in home mean SBP from randomization to week 12	Adverse events (AEs) leading to discontinuation of trial medication from baseline to week 12	1500, ≥95% for each of the three comparisons

GMRx2 PCT	Randomized, double-blind, placebo-controlled, parallel-group, international multi-center	Adults on ≤2 BP-lowering drugs, adhering to and tolerating placebo run-in and post run-in home SBP 130-154 mmHg	Receiving 2 or more BP-lowering drugs. Contraindication to placebo run-in or any of the randomized medications	2:2:1 ratio to GMRx2 dose 1, GMRx2 dose 2 or placebo	Intervention 1: GMRx2 dose 1 for 4 weeks Intervention 2: GMRx2 dose 2 for 4 weeks Comparator: Placebo for 4 weeks	4 weeks + 4 weeks safety follow up	Difference in change in home mean SBP from randomization to week 4	AEs leading to discontinuation of trial medication from baseline to week 4	250, >90% for GMRx2 vs placebo, and >80% for GMRx2 dose 1 vs GMRx2 dose2


**Table 4:** GMRx2 Program Steering Committee.


NAME	AFFILIATION	ROLE

Professor Paul Whelton	Tulane University, New Orleans, USA	Steering Committee Member (Chair)

Professor William Cushman	University of Tennessee Health Science Center, USA	Steering Committee Member

Professor Asita de Silva	University of Kelaniya, Sri Lanka	Steering Committee Member

Professor Diederick Grobbee	University Medical Center Utrecht, Utrecht University, The Netherlands	Steering Committee Member

Professor Krzysztof Narkiewicz	Medical University of Gdańsk, Poland	Steering Committee Member

A/Professor Dike Ojji	University of Abuja, Nigeria	Steering Committee Member

Professor Suzanne Oparil	University of Alabama at Birmingham, USA	Steering Committee Member

Professor Neil Poulter	Imperial College, London, UK	Steering Committee Member

Professor Markus Schlaich	The University of Western Australia, Australia	Steering Committee Member

A/Professor Wilko Spiering	University Medical Center Utrecht, Utrecht University, The Netherlands	Steering Committee Member

Professor Bryan Williams	University College, London, UK	Steering Committee Member

Professor Jackson T Wright Jr	University Hospitals Cleveland Medical Center, Case Western Reserve University, USA	Steering Committee Member

Professor Anthony Rodgers*	The George Institute for Global Health, University of New South Wales, Australia	Steering Committee Member, Academic Coordinating Center

Dr Abdul Salam*	The George Institute for Global Health, University of New South Wales, India	Steering Committee Member, Academic Coordinating Center

Professor Aletta E Schutte*	The George Institute for Global Health, University of New South Wales, Australia	Steering Committee Member, Academic Coordinating Center

Professor Gian Luca Di Tanna*	The George Institute for Global Health, University of New South Wales, Australia	Academic Coordinating Center


* Non-voting members.

**Table 5:** GMRx2 Data and Safety Monitoring Board.


NAME	AFFILIATION	ROLE

Professor Lawrence Appel	Johns Hopkins University, Bal, USA	Chair

Professor Mark Espeland	Wake Forest University, NC, USA	Member

Professor Michael Weber	State University of New York, NY, USA	Member

Professor Gian Luca Di Tanna	University of Applied Sciences and Arts of Southern Switzerland, Lugano, Switzerland	Blinded statistician

Chris Gianacas	The George Institute for Global Health, Sydney, Australia	Blinded statistician

Xiaoqiu (Julia) Liu	The George Institute for Global Health, Sydney, Australia	Unblinded statistician

Michelle Leroux	Toronto, Canada	Executive Secretary


### Active-controlled trial

**Figure 2:**
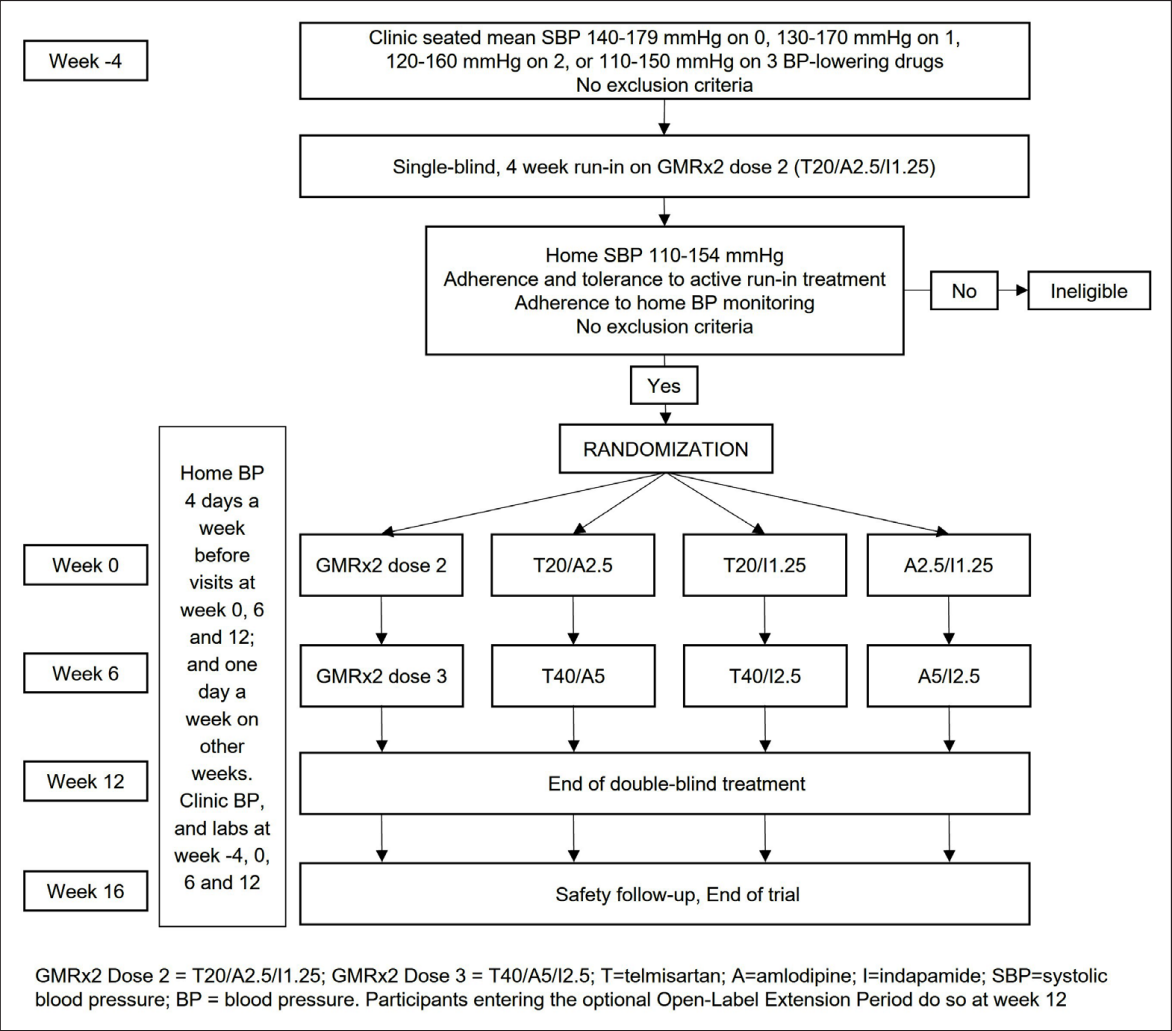
Schema for trial comparing GMRx2 with each dual combination (GMRx2 ACT).

The main objective of this trial is to assess the contribution of each GMRx2 component drug to its overall effects, by assessing the efficacy and safety of GMRx2 compared to each of the three possible dual combinations of the component drugs. The key eligibility criteria are individuals with hypertension currently receiving 0–3 medications, for whom there is uncertainty about treatment with GMRx2 vs dual combinations. The design is summarized in Figure 2. The run-in period will assess participant adherence to trial medication and trial procedures, hence maximizing the likelihood of participant follow-up and complete data collection. The double-blind period will assess the efficacy and safety of GMRx2 dose versions 2 and 3, sequentially, compared to the corresponding three possible dual combinations of the component drugs. The duration of six weeks for each treatment period will allow comparison of the maximal effects of treatment. The safety follow-up period will assess safety over 4 weeks following switching from randomized trial medication to open label usual care treatment.

Participants who adhere to GMRx2 dose 2 during the run-in period and have an average home SBP 110–154 mmHg at the end of run-in period. Participants are being recruited from Australia, the Czech Republic, New Zealand, Poland, Sri Lanka, the United Kingdom and the USA. The primary outcome is the difference in change of home SBP from randomization to week 12 (averaged over the preceding week). Randomization was completed with 1385 participants in May 2023, which was >90% of the original 1500 planned, with the steering committee recommending a reduced sample size, without any reference to unblinded data, given the likelihood of adequate power and unexpected problems with drug supply due to pandemic-related supply chain issues. This sample size will provide over 97% power to detect a minimum clinically significant difference of 3 mmHg in mean home seated level of SBP for each of the three comparisons of GMRx2 vs dual therapy, assuming a common standard deviation in SBP of 11 mmHg and a correlation coefficient of 0.4. The overall power for all three comparisons will therefore be > 90%. Previous trials have demonstrated an additional reduction in office SBP of approximately 5 mmHg [42].

### Placebo-controlled trial

**Figure 3:**
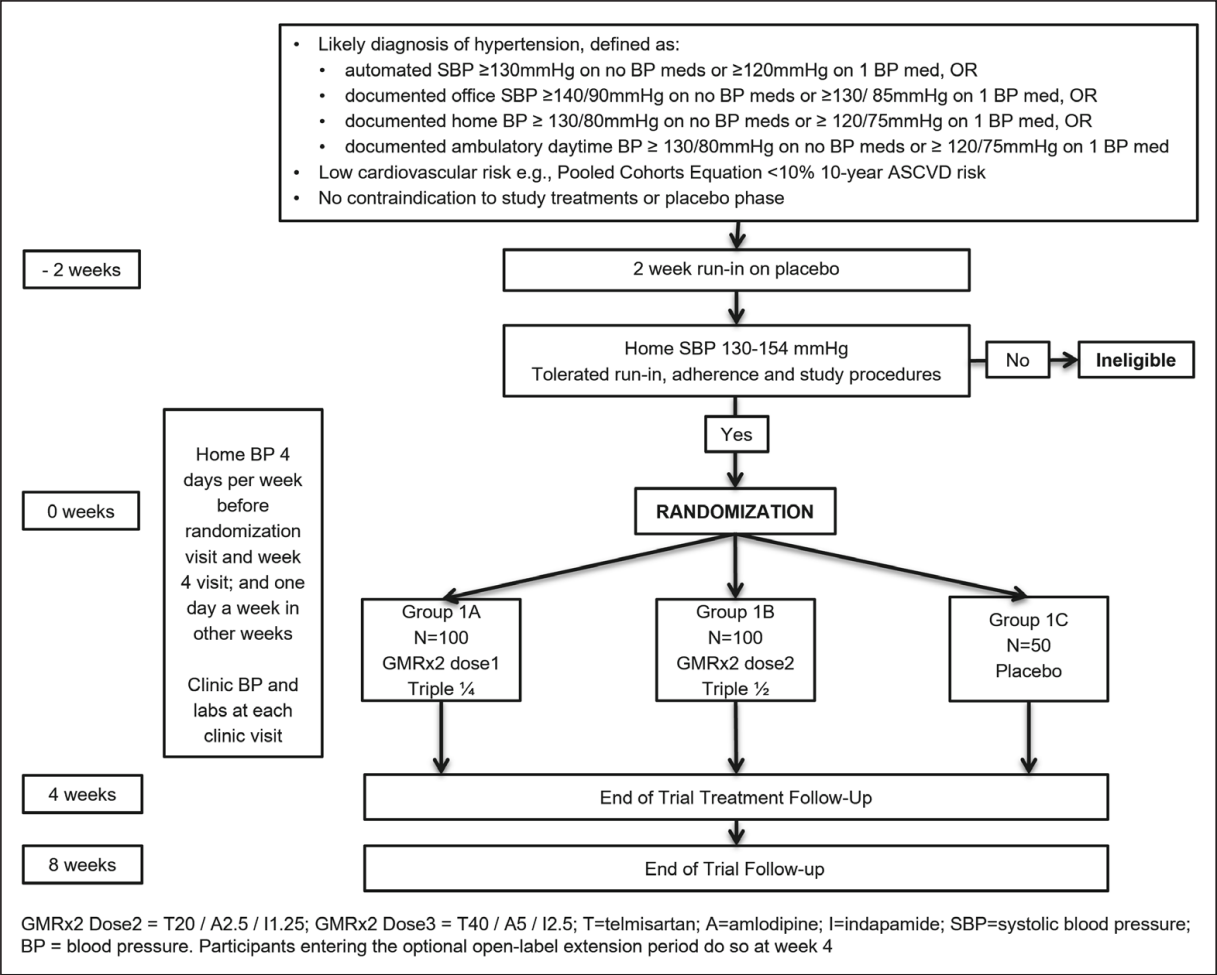
Schema for trial comparing GMRx2 with placebo (GMRx2 PCT).

The main objective of this trial is to investigate the efficacy and safety of GMRx2 compared to placebo. Placebo controlled trials provide important evidence on the full effects of treatment, including both efficacy and safety. Without such evidence it would be difficult to assess the full degree of efficacy, or to reliably attribute the causality of any adverse effects observed. The key eligibility criteria are individuals with hypertension currently receiving 0–1 medications and low estimated cardiovascular risk (e.g., Pooled Cohorts Equation <10% ten-year risk), for whom there is uncertainty about treatment with GMRx2 vs. placebo. The design is summarized in Figure 3. The placebo run-in period will assess each participant’s adherence to trial procedures and allow confirmation of untreated mean BP levels, hence enhancing the power of the trial to detect a difference between GMRx2 and placebo. The double-blind period will assess the efficacy and safety of GMRx2 dose 1 and GMRx2 dose 2 compared to placebo. The safety follow-up period will assess safety over four weeks following switching from randomized trial medication to open label usual care treatment.

Participants who tolerate placebo and whose average home SBP is between 130–154 mmHg after a two-week run-in period on placebo are eligible for randomization. Participants were recruited from Australia, Nigeria, Sri Lanka and the USA. The primary outcome is difference in change in home SBP from randomization to week 4 (averaged over the week preceding the final clinic visit). The trial sample size of 250 randomized participants (100 each to GMRx2 strength 1 and 2, and 50 to placebo) is expected to provide 90% power to detect a difference of at least 9 mmHg in mean home SBP for each of the active drug comparisons vs placebo, assuming a common standard deviation of 11 mmHg.

#### Open-label extension of active and placebo-controlled trial

An open-label extension period of the above two trials will be conducted in some sites, to assess the efficacy and safety of GMRx2 over a one-year period. Willing participants on ≤3 BP-lowering drugs, being adherent to the trial procedures will be consented to be switched at the end of the double-blind period to open label GMRx2. Follow-up visits for the open-label extension study will be scheduled at weeks 2 and 4, given the evidence that almost all the BP-lowering effects of treatment will accrue within two weeks [43], hence providing an opportunity for the study to assess the effects of an evidence-based, accelerated titration schedule. Subsequent follow-up visits will be conducted at week 12, 28, and 40. Participants will continue to monitor home BP, with uptitration for those with home BP > 130/80 mmHg: GMRx2 strength 1 à GMRx2 strength 2 → GMRx2 strength 3 → GMRx2 strength 3 plus telmisartan 40 mg/amlodipine five mg → GMRx2 strength 3 plus telmisartan 40 mg/amlodipine 5 mg plus spironolactone 25 mg. Each titration step was chosen to provide an estimated additional SBP reduction of at least 5 mmHg, in keeping with the strategy of avoiding titration steps that only confer additional average SBP reductions of 1–3 mmHg, such as doubling the dose of a single drug [10,15,42].

### Other studies

#### Pharmacokinetic studies

The GMRx2 development program also includes pharmacokinetic studies [44,45]. The first trial aimed to evaluate pharmacokinetic drug interactions between telmisartan, amlodipine and indapamide and GMRx2. The trial design was a single center, open, randomized, single-dose, two-period, four-treatment, two-sequence, crossover study with a washout period between doses of at least 14 days for Cohort 1 (telmisartan) and Cohort 3 (indapamide), and 28 days for Cohort 2 (amlodipine). In total, 122 healthy volunteers were enrolled and randomized. The second trial aims to evaluate the effect of food on bioavailability (AUC and Cmax) of GMRx2. It is a single center, open, randomized, single-dose, two-period, two-treatment (fed vs. fasting), two-sequence, crossover study with a washout period between doses of at least 28 days. Performed where?

#### The delivery of optimal blood pressure control in Africa (VERONICA) – Nigeria trial

VERONICA-Nigeria is an investigator-initiated trial being conducted in Black Africans that is comparing a triple therapy SPC-based treatment protocol with treatment based on the Nigeria hypertension guidelines to determine the effectiveness, safety, and feasibility of the SPC for control of high BP in Nigeria. Eligibility is restricted to Black African adults who are untreated or on one BP-lowering medication for ≥2 weeks with clinic BP 140–179 mmHg and/or DBP 90–109 mmHg. The trial compares treatment with an accelerated, simplified, GMRx2-based regimen to the Nigeria hypertension treatment protocol, and the primary outcome is difference in change in home SBP from randomization to month 6. A total of 300 participants are planned, with outcomes expected in late 2023.

#### Triple therapy prevention of recurrent intracerebral disease events trial (TRIDENT)

TRIDENT is an investigator initiated and conducted, multicenter, international, double-blinded, placebo-controlled, parallel-group, randomized trial designed to determine the efficacy of more intensive BP control with a low-dose SPC (GMRx2 dose 2) strategy in addition to standard of care, on stroke recurrence in patients with a history of acute stroke due to intracerebral hemorrhage [46]. Eligible participants are adults with a history primary intracerebral hemorrhage, SBP of 130–160 mmHg, and no contraindication to the randomization of GMRx2-dose version 2 or placebo in addition to any existing BP lowering regimen. The primary outcome is time to first occurrence of recurrent stroke. A total of 1500 randomized participants are planned with a mean duration of 3 years follow-up.

## Discussion

Low-dose triple SPCs have the potential to significantly improve BP control by providing improved efficacy and adherence, and reduced therapeutic inertia with fewer adverse effects compared to current approaches. However, to date there is limited clinical research in this area, and no low-dose triple SPCs on the market globally. The GMRx2 clinical development aims to address these gaps, assessing efficacy and tolerability, with two pivotal regulatory approval trials, and a series of investigator-initiated studies.

The phase III and pharmacokinetics trial program described in this manuscript has been approved by the FDA to potentially support an indication for the treatment of hypertension, including initial treatment. Regulatory approval will also be sought in other markets globally, including importantly low- and middle- income countries (LMICs). Globally there is a dearth in availability and affordability of SPCs in LMICs, where more effective treatment is most needed [47,48]. For example, BP levels are now among the highest globally in Sub-Saharan Africa, where over 200 million people are projected to have hypertension by 2030. Furthermore, hypertension is the leading cause of the rapidly increasing burden of noncommunicable diseases, and less than one in seven with hypertension achieve adequate control [2,49,50]. Market access is also planned in high income settings outside of the USA, but this may be delayed in Europe, given the current requirements for additional studies [51]. The majority of SPC products in Europe are approved only on the basis of bioequivalence studies, and such approvals lead to a ‘straight substitution’ indication (i.e., for use only in people already stabilized on the same drugs at the same doses). This is not possible or appropriate for novel low-dose combinations, developed to help address poor adherence and treatment inertia.

Following regulatory approval of GMRx2, additional studies and implementation research will be required to further delineate the role and cost-effectiveness of GMRx2 compared to current usual care modalities in different patient populations and geographies. Further indications will also be explored such as the prevention of stroke in people with intracerebral hemorrhage [46] and the prevention of dementia [52]. A key issue will be reimbursement in HIC markets, availability and affordability. Most importantly research will be conducted to inform access and appropriate scale-up in disadvantaged populations, where the burden of high BP is highest, and reliable access to affordable, effective therapies is lowest [53].

## Abbreviations

ACEI: Angiotensin converting enzyme inhibitor

ARB: angiotensin receptor blocker

BP: blood pressure

CCB: calcium channel blocker

FDA: United States Food and Drug Administration

LMIC: low- and middle- income country

RCT: randomized controlled trial

SPC: single pill combination

SBP: systolic blood pressure

USA: United States of America
